# Effects of Remimazolam and Propofol on Ca^2+^ Regulation by Ryanodine Receptor 1 with Malignant Hyperthermia Mutation

**DOI:** 10.1155/2021/8845129

**Published:** 2021-01-04

**Authors:** Tomoyuki Watanabe, Hirotsugu Miyoshi, Yuko Noda, Soshi Narasaki, Atsushi Morio, Yukari Toyota, Hiroshi Kimura, Keiko Mukaida, Toshimichi Yasuda, Yasuo M. Tsutsumi

**Affiliations:** ^1^Department of Anesthesiology and Critical Care, Hiroshima University, Hiroshima 734-8551, Japan; ^2^Department of Anesthesiology, Hiroshima Prefectural Rehabilitation Center, Higashihiroshima 739-0036, Japan

## Abstract

**Background:**

We investigated the potential safety of remimazolam and propofol in malignant hyperthermia- (HM-) susceptible patients using ryanodine receptor 1- (RYR1-) expressing human embryonic kidney- (HEK-) 293 cells.

**Methods:**

We compared the enhanced responsiveness of HEK-293 cells expressing wild-type *RYR1* with that of mutant *RYR1* to caffeine following perfusion with remimazolam or propofol. Furthermore, we investigated whether RYR1 enhanced the responsiveness of cells to remimazolam or propofol and compared the median effective concentration (EC_50_; i.e., the concentration required to reach half-maximal activation) using an unpaired two-tailed *t*-test while a *P* < 0.05 was considered significant.

**Results:**

Remimazolam and propofol did not promote the caffeine-induced increase in intracellular Ca^2+^ levels in HEK-293 cells expressing mutant RYR1 even with exposure to approximately 100-fold the clinically used concentration. In wild-type RYR1, EC_50_ values of remimazolam following refusion vs. nonperfusion were 2.86 mM vs. 2.75 mM (*P* = 0.76) while for propofol perfusion vs. nonperfusion, they were 2.76 mM vs. 2.75 mM, respectively (*P* = 0.83). In mutant RYR1, EC_50_ values of remimazolam refusion vs. nonperfusion were 1.58 mM vs. 1.71 mM, respectively (*P* = 0.63) while for propofol perfusion vs. nonperfusion, they were 1.65 mM vs. 1.71 mM, respectively (*P* = 0.73). Remimazolam and propofol increased intracellular Ca^2+^ levels in a concentration-dependent manner, but the effect was not enhanced by RYR1. EC_50_ values of remimazolam with non-RYR1 vs. wild-type RYR1 were 1.00 mM vs. 0.92 mM, respectively (*P* = 0.91) while those of propofol were 1.09 mM vs. 1.05 mM, respectively (*P* = 0.84).

**Conclusions:**

The increase in intracellular Ca^2+^ concentration caused by remimazolam or propofol was not considered an RYR1-mediated reaction. We conclude that remimazolam and propofol can be safely used as an anesthetic in MH-susceptible patients with *RYR1*-mutation without causing MH and may be safely substituted for an MH-triggering anesthetic when RYR1-mediated MH occurs.

## 1. Introduction

Malignant hyperthermia (MH; OMIM #145600) is an autosomal dominant pharmacogenetic disorder and one of the serious complications caused by exposure of MH-susceptible patients to volatile anesthetics [1, 2]. The pathogenesis of this disease is dysregulation of intracellular Ca^2+^ in skeletal muscle cells while dysfunction of the ryanodine receptor 1 (RYR1), a Ca^2+^ release channel in the sarcoplasmic reticulum of skeletal muscle cells, is a known etiological factor [3]. In patients who are MH-susceptible, RYR1 dysfunction dramatically enhances Ca^2+^ release from the sarcoplasmic reticulum into the cytoplasm, increasing cytoplasmic Ca^2+^ concentration only following exposure to triggering agents [4]. Furthermore, elevated cytoplasmic Ca^2+^ concentration in skeletal muscle promotes hypermetabolism, resulting in clinical symptoms such as respiratory/metabolic acidosis, elevated body temperature, and muscle rigidity. In anesthetic management of MH, it is important for genetically susceptible patients to avoid exposure to MH-triggering anesthetics. Therefore, the genetic susceptibility of patients should be determined before the use of select anesthetics such as midazolam and propofol that do not usually cause MH [5, 6]. Remimazolam is an ultrashort acting intravenous benzodiazepine sedative/anesthetic used to induce sedation in the US, EU, and China [7]. It is also used as a general anesthetic in Japan [8, 9], and it is necessary to determine whether it causes MH.

To elucidate the regulatory effects of remimazolam and propofol on intracellular Ca^2+^, we investigated whether exposure to these agents promotes the responsiveness of various *RYR1*-expressing human embryonic kidney 293 (HEK-293) cells to caffeine, which is an agonist of RYR1. In addition, we also calculated the median effective concentration (EC_50_) of remimazolam and propofol and compared the changes in their values with and without RYR1 in HEK-293 cells.

## 2. Materials and Methods

### 2.1. Plasmid-Cloning DNA and Cell Culture Reagents

Full-length rabbit skeletal muscle *RYR1*/pcDNA (rabbit-*RYR1*/pcDNA) was a generous gift from David H. MacLennan (University of Toronto). Remimazolam was a generous gift from PAION Deutschland GmbH (Aachen, Germany) and Mundipharma Japan (Tokyo, Japan). The HEK-293 Tet system-approved cell line was purchased from Clontech Laboratories (Mountain View, CA, USA) while the restriction enzymes were from Takara Bio (Otsu, Japan). Caffeine and other chemicals were from Wako Pure Chemical Industries (Osaka, Japan).

### 2.2. Preparation of RyR1/pTRE-Tight-BI-AcGFP (RyR1/pBI)

The pTRE-Tight-BI-AcGFP (pBI, Clontech Laboratories) vector allowed the inducible expression of a reporter, green fluorescent protein (AcGFP1) with the gene of interest. Full-length rabbit *RYR1* cDNA was removed from rabbit *RYR1*/pcDNA using the restriction enzymes XbaI and HindIII. Then, the rabbit *RYR1* cDNA was inserted into the NheI-HindIII site of pTRE-Tight-BI-AcGFP using the Takara DNA ligation kit (LONG, Takara Bio).

### 2.3. Mutagenesis

The regions of interest, which were the C-terminal, central, and N-terminal of *RYR1*, were removed from the *RYR1*/pBI using restriction enzymes. Each fragment was inserted into the pBluescript II KS (+) vector (Stratagene Cloning Systems, La Jolla, CA, USA) and used as a template for mutagenesis. Mutagenesis was performed using the QuikChange II XL site-directed mutagenesis kit (Agilent Technologies, Santa Clara, CA, USA). We designed the following three mutation primers, which have been detected in MH patients: 5′-GAT GCT GGC CAA CAT GGT GGA GGC TGG CGT-3′ for p.Thr84Met, 5′-GTT AAC GGC GAG AGA GTG GAG AAC-3′ for p.Ser2345Arg, and 5′-CGT GGG CGT CCG GAC TGG CGG AGG-3′ for p.Ala4894Thr. After mutagenesis, we sequenced pBluescript II KS (+) using an ABI PRISM 3100 genetic analyzer (Applied Biosystems, Foster City, CA, USA) using a BigDye Terminator v3.1 cycle sequence kit (Applied Biosystems). To construct the vectors for expressing the mutated *RYR1* gene, the mutated fragments were removed from the pBluescript II KS (+) plasmid using the restriction enzymes *Nhe*I and *Kpn*I for p.Thr84Met, *Spe*I and *Bsi*WI for p.Ser2345Arg, and *Cla*I and *Hind*III for p.Ala4894Thr and ligated into *RYR1*/pBI.

### 2.4. Prediction of Deleteriousness

The deleteriousness of the three *RYR1* mutations, p.Thr84Met, p.Ser2345Arg, and p.Ala4894Thr, was predicted using predictive application tools, Combined Annotation-Dependent Depletion (CADD) [10], MutationTaster [11], and PolyPhen-2 [12].

### 2.5. Cell Culture

The HEK-293 cells were maintained in Dulbecco's modified Eagle's medium (DMEM; Invitrogen, Carlsbad, CA, USA) supplemented with 10% Tet system-approved fetal bovine serum (FBS; Clontech Laboratories), 100 U/mL penicillin (Sigma-Aldrich, St. Louis, MO, USA), and 100 mg/mL streptomycin (Sigma-Aldrich) at 37°C in an atmosphere of 5% CO_2_.

### 2.6. DNA Transfection

HEK-293 cells (1 × 10^5^ cells per dish) were subcultured in 35 mm poly-l-lysine-coated glass-bottomed dishes (Matsunami Glass Ind., Osaka, Japan) for 24 hours. DNA transfection was performed using the FuGENE HD transfection reagent (Roche Applied Science, Indianapolis, IN, USA) 72 hours before measurement of Ca^2+^ release.

### 2.7. Protein Extraction and Immunoblot Analysis

We incubated the *RYR1* transfected (wild-type or mutant) and untransfected HEK-293 cells in a 100 mm dish until they were confluent. Protein lysates were separated using sodium dodecyl sulfate-polyacrylamide gel electrophoresis (SDS-PAGE) with 10% polyacrylamide precast gels (Bio-Rad Laboratories, Hercules, CA, USA) and transferred onto polyvinylidene difluoride membranes by electroelution. Membranes were blocked in 20 mM Tris-buffered saline (TBS) plus Tween (0.1%) containing 2% skim milk and incubated with monoclonal primary antibodies (RYR1 (D4E1), #8153, Cell Signaling Technology, Danvers, MA, USA) overnight at 4°C. Immunolabeled blots were visualized using horseradish peroxidase-conjugated secondary antibodies (Santa Cruz Biotechnology, Dallas, TX, USA) and visualized using an enhanced chemiluminescence reagent (Bio-Rad Laboratories) [13].

### 2.8. Ca^2+^ Fluorescence Measurements

#### 2.8.1. Experiment 1: Reactivity to Caffeine

As previously described, HEK-293 cells transfected with *RYR1*/pBI (wild-type) or one of the three *RYR1*/pBI mutants (p.Thr84Met [14], p.Ser2345Arg [15], or p.Ala4894Thr [16]) were washed with Hank's balanced salt solution (HBSS) containing 130 mM sodium chloride (NaCl), 5.4 mM potassium chloride (KCl), 20 mM HEPES, 2.5 mM calcium chloride (CaCl_2_), 1 mM magnesium chloride (MgCl_2_), and 5.5 mM glucose (pH 7.4). The cells were loaded with 5.0 *μ*M Fura-2 AM (Dojindo Molecular Technologies, Tokyo, Japan) in HBSS for 1 hour at 37°C and then washed with HBSS. Measurements were performed after perfusion with HBSS for 15 minutes. Next, the cells were excited at 490 nm and fluorescence emissions of AcGFP were observed at 510 nm to identify AcGFP-expressing cells, which were regarded as successfully transfected [17]. The AcGFP-positive cells were excited alternately at 340 and 380 nm, and fluorescence emissions of Fura-2 AM were observed at 510 nm using a fluorescence microscope (Nikon, Tokyo, Japan) at 5-second intervals to evaluate intracellular Ca^2+^ changes. Images were acquired using a cooled high-speed digital video camera (ORCA-AG; Hamamatsu Photonics, Hamamatsu, Japan).

HBSS samples containing increasing concentrations of caffeine were added to one side of the culture dish and aspirated from the opposite side for 2 minutes at a rate of 1.2 mL/min at 37°C. Each solution was washed out 2 minutes before the addition of the next higher concentration of caffeine. Changes in Fura-2 AM fluorescence induced by various caffeine concentrations (0.31, 0.62, 1.25, 2.5, 5, 10, and 20 mM) were measured, and the 340/380 nm signal ratio was calculated using a Ca^2+^ imaging system (Aquacosmos 2.5; Hamamatsu Photonics) within 60 minutes after washing out the excess Fura-2 AM. The dish was rinsed with HBSS for 3 minutes before the next concentration was applied.

#### 2.8.2. Experiment 2: Reactivity to Caffeine with Propofol or Remimazolam Perfusion

For this experiment, remimazolam (546 *μ*M) or propofol (100 *μ*M) was added to HBSS, which was then continuously refluxed. Then, the change in intracellular Ca^2+^ concentration when caffeine was applied at increasing concentrations from 0.31 to 20 mM was measured using the same method used in Experiment 1.

#### 2.8.3. Experiment 3: Reactivity to Remimazolam or Propofol

This experiment was similar to Experiment 1 except that the changes in Fura-2 AM fluorescence induced by various concentrations of remimazolam (26, 52, 105, 209, 418, 836, 1674, 3347, and 5020 *μ*M) or propofol (156, 312, 625, 1250, 2500, and 5000 *μ*M), rather than caffeine, were measured. The HEK-293 cells used in this experiment were those in which the *RYR1* gene was not mutated (i.e., *RYR1* untransfected) and those in which the wild-type *RYR1* was mutated.

### 2.9. Data Analysis

To trace the concentration-response curves of caffeine, remimazolam, and propofol, the data were normalized to the maximal response of each cell. Then, the EC_50_ of caffeine was calculated from the concentration-response curves and all data were analyzed using PRISM 7.0 software (GraphPad Software, San Diego, CA, USA). The EC_50_ was measured in each cell and used as a summary measure.

In Experiment 1, we compared the EC_50_ value of cells expressing each mutant RYR1 with that of cells expressing wild-type RYR1. In Experiment 2, we compared the EC_50_ value of cells expressing wild-type RYR1 or each mutant RYR1 perfused with remimazolam or propofol. In Experiment 3, the EC_50_ values of remimazolam and propofol were compared in cells with and without expression of wild-type RYR1.

Statistical significance between EC_50_ values was calculated using an unpaired two-tailed *t*-test, and a *P* value = 0.05 was considered statistically significant. The sample size was calculated by Gpower software to require 10 samples for each group. Since we usually perform the experiment 3 times to confirm the reproducibility of the experiment, the number of samples may exceed the sample size. When the number of samples was more than 10 and the *P* value was less than 0.05, we calculated Cohen's d to confirm the effect size.

## 3. Results

### 3.1. Confirmation of RYR1 Expression

**Figure 1:**
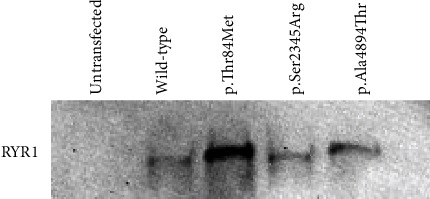
Western blot image. Western blot image confirmed the presence of ryanodine receptor 1 (RYR1) protein in the human embryonic kidney- (HEK-) 293 cells transfected with wild-type or mutant RYR1, p.Thr84Met, p.Ser2345Arg, or p.Ala4894Thr.

We confirmed the expression of RYR1 in cells transfected with wild-type or mutant RYR1 and its absence in untransfected cells using immunoblotting (Figure 1).

### 3.2. Transfection Efficiency

The proportion of AcGFP-expressing cells was approximately 70%, and there was no obvious difference in transfection efficiency between the wild-type and mutated RYR1 constructs [17].  Table 1 shows the Fura-2 ratio (340/380 nm) of HEK-293 cells expressing wild-type or mutation RYR1 at resting state and at response to 10 mM caffeine and 20 mM caffeine. Since the values of the Fura-2 ratio at resting represent the leakage of Ca^2+^ from the sarcoplasmic reticulum (SR) by RYR1, the results we present in Table 1 show that there is no significant difference in the expression levels of wild-type RYR1 and mutated RYR1. Similarly, since the Fura-2 ratio in response to 10 mM and 20 mM caffeine represents the Ca^2+^ storage of SR, the results indicate that there is no significant difference in Ca^2+^ storage between wild-type and mutant RYR1-expressing HEK-293 cells. The fact that there is no difference between the response to 10 mM caffeine and the response to 20 mM caffeine at the Fura-2 ratio of each RYR1 indicates that these are the maximum amounts of Ca^2+^ stores released from SR.

### 3.3. Evaluation of Deleteriousness of Mutations

The results of *in silico* prediction of the deleteriousness effect on the function of protein products of the three *RYR1* mutations assessed using CADD, MutationTaster, and PolyPhen-2 are shown in Table 2. All three *RYR1* mutations had high CADD scores (>20) and were predicted to be “disease causing” and “possibly damaging” by the MutationTaster and PolyPhen-2, respectively.

### 3.4. Representative Reaction

**Figure 2:**
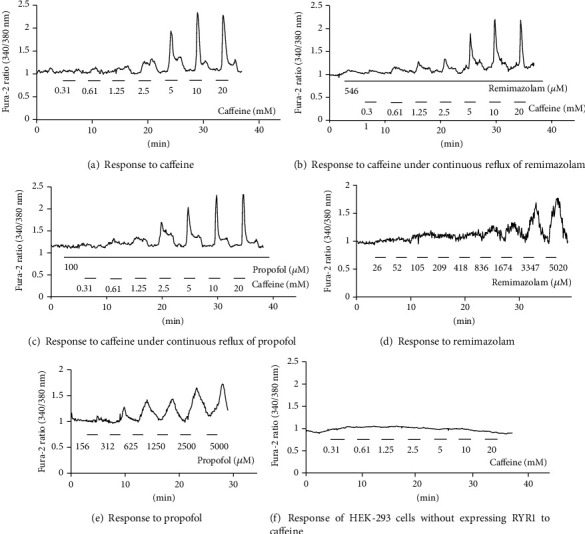
Representative Ca^2+^ release response of wild-type ryanodine receptor 1- (RYR1-) transfected human embryonic kidney- (HEK-) 293 cells or non-RYR1-transfected HEK-293 cells. Representative traces of agonist-induced Ca^2+^ release by HEK-293 cells transfected with wild-type pcRYR1 cDNA or without any pcRYR1 cDNA. The vertical axis represents the Fura-2 ratio (340/380 nm), i.e., intracellular Ca^2+^ release, and the horizontal axis represents time (minutes). The response of wild-type RYR1 expressing HEK-293 cells to (a) caffeine, (b) caffeine with continuous reflux of remimazolam (546 *μ*M, 100-fold higher than clinical dose), (c) caffeine with continuous reflux of propofol (100 *μ*M, 100-fold higher than the clinical dose), (d) remimazolam (increasing concentrations up to 5020 *μ*M), and (e) propofol (increasing concentrations up to 5000 *μ*M). The response of HEK-293 cells without expressing RYR1 to caffeine (f). No increase in intracellular Ca^2+^ levels for caffeine was observed in HEK-293 cells without expressing RYR1.

Representative Ca^2+^ release responses of wild-type *RYR1* cDNA-transfected HEK-293 cells or non-*RYR1* cDNA-transfected HEK-293 cells are shown in Figure 2. In HEK-293 cells expressing wild-type RYR1, intracellular Ca^2+^ levels increased in a concentration-dependent manner for caffeine. Similarly, intracellular Ca^2+^ levels increased in a concentration-dependent manner for remimazolam and propofol. An increase in intracellular Ca^2+^ levels for caffeine was not observed in HEK-293 cells without expressing RYR1.

### 3.5. Functional Analysis

**Figure 3:**
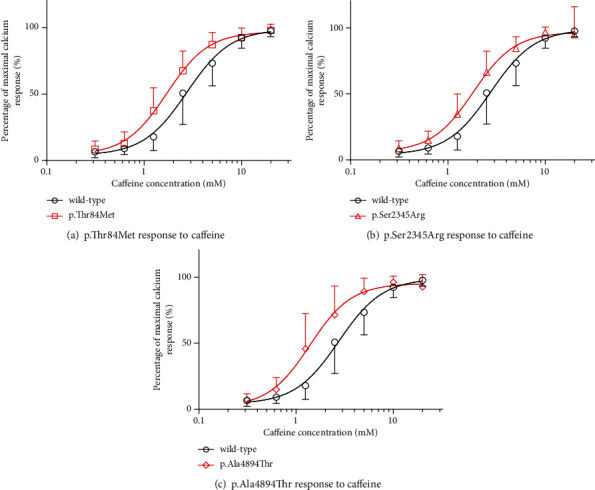
Caffeine concentration-dependent Ca^2+^ release in human embryonic kidney- (HEK-) 293 cells transfected with wild-type or mutant ryanodine receptor 1 (RYR1). Caffeine dose-response of Ca^2+^ release in HEK-293 cells transfected with wild-type or mutant RYR1. Data were normalized to the maximal response of each cell type. The vertical axis represents the percentage of maximal Ca^2+^ response, and the horizontal axis represents caffeine concentration (mM). Data are means ± SD in mutants and wild-type. Circles and other symbols represent the wild-type and mutant RYR1-transfected groups. The response of (a) wild-type RYR1 and p.Thr84Met RYR1-transfected cells, (b) wild-type RYR1 and p.Ser2345Arg RYR1-transfected cells, and (c) wild-type RYR1 and p.Ala4894Thr RYR1-transfected cells to caffeine. Curves of all mutant RYR1-transfected cells were shifted leftward relative to those of wild-type RYR1-transfected cells.

The caffeine concentration-response curves of cells transfected with the wild-type and mutant (p.Thr84Met, p.Ser2345Arg, or p.Ala4894Thr) *RYR1* are shown in Figure 3. The curves of all mutant *RYR1*-transfected cells were shifted to the left relative to those of the wild-type *RYR1*-transfected cells. The EC_50_ values were significantly lower in all mutant *RYR1*-transfected cells than in wild-type *RYR1*-transfected cells (Table 3).

**Figure 4:**
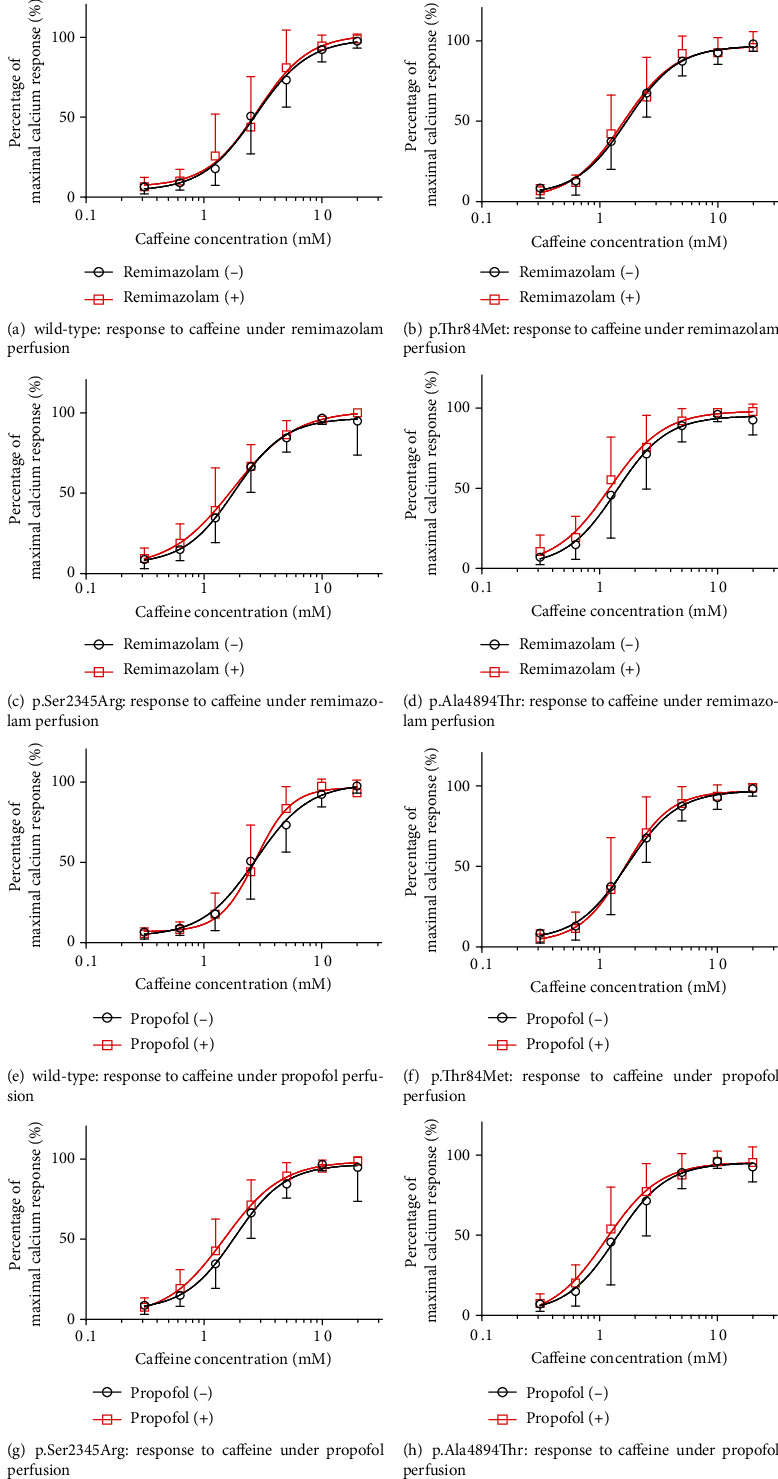
Caffeine concentration-dependent Ca^2+^ release in human embryonic kidney (HEK-293) cells transfected with wild-type or mutant ryanodine receptor 1 (RYR1) perfused with remimazolam or propofol. Caffeine-induced Ca^2+^ release in HEK-293 cells transfected with wild-type or mutant RYR1 perfused with remimazolam or propofol. Data were normalized to the maximal response of each cell type. Vertical and horizontal axes represent percentage maximal Ca^2+^ response and caffeine concentration (mM). Data are means ± SD in cells perfused with or without remimazolam or propofol. Squares and circles represent remimazolam or propofol perfusion and nonperfused groups, respectively. (a–d) Remimazolam and (e–h) propofol perfused cells. Response of (a) wild-type, (b) p.Thr84Met, (c) p.Ser2345Arg, (d) p.Ala4894Thr, (e) wild-type, (f) p.Thr84Met RYR1, (g) p.Ser2345Arg, and (h) p.Ala4894Thr. Curves of all mutant and wild-type RYR1-transfected cells did not show a clear leftward shift under perfusion of remimazolam or propofol.

Figure 4 shows the caffeine concentration-response curve of cells transfected with the wild-type and mutant (p.Thr84Met, p.Ser2345Arg, or p.Ala4894Thr) *RYR1* and perfused with remimazolam or propofol. The curves of all mutant and wild-type *RYR1*-transfected cells did not show a clear leftward shift following perfusion with remimazolam or propofol. The EC_50_ values of caffeine in wild-type and all mutant *RYR1*-transfected cells were not significantly different from those of cells with or without remimazolam and propofol perfusion (Table 4).

**Figure 5:**
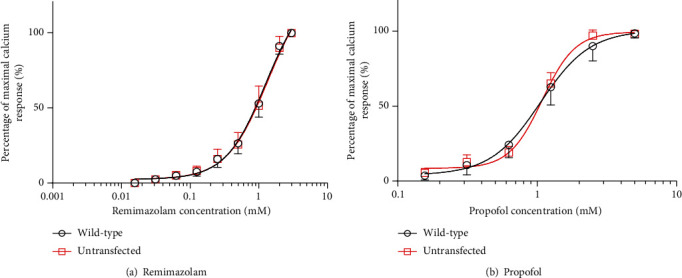
Remimazolam and propofol concentration-dependent Ca^2+^ release by human embryonic kidney- (HEK-) 293 cells transfected with or without wild-type ryanodine receptor 1 (RYR1). Remimazolam and propofol concentration-response curves of RYR1-untransfected cells and wild-type RYR1-transfected cells are shown. Data were normalized to the maximal response of each cell type. The vertical axis represents percentage maximal Ca^2+^ response, and the horizontal axis represents remimazolam or propofol concentration (mM). Data are means ± SD in nontransfected and wild-type RYR1. Circles and squares represent wild-type RYR1 and untransfected groups, respectively. Dose-response curves of (a) remimazolam and (b) propofol. Curves showed no significant leftward shift in wild-type RYR1-transfected cells compared to untransfected RYR1 cells.

The concentration-response curves of remimazolam and propofol showed no significant leftward shift in cells transfected with wild-type *RYR1* compared to untransfected cells (Figure 5). The EC_50_ values of remimazolam or propofol in wild-type *RYR1*-transfected and untransfected cells were not significantly different (Table 5).

## 4. Discussion

In this study, we evaluated whether exposure to remimazolam or propofol enhances the caffeine-induced elevation of intracellular Ca^2+^ levels in HEK-293 cells expressing wild-type or mutant RYR1. We found that the response to caffeine was not enhanced by either propofol or remimazolam in HEK-293 cells expressing wild-type or mutant RYR1. Furthermore, we also evaluated whether the elevation of intracellular Ca^2+^ by remimazolam or propofol was promoted by the presence or absence of RYR1. The result showed that the response to remimazolam and propofol was not enhanced more in HEK-293 cells expressing wild-type RYR1 than those not expressing wild-type RYR1. Based on the study results, we conclude that remimazolam and propofol are unlikely to cause RYR1-mediated MH.

We used 240 *μ*g/mL of remimazolam, which corresponded to 546 *μ*M and was 100-fold higher than the plasma concentration following a clinically used dose [18]. Similarly, propofol was used at 17.8 *μ*g/mL, which corresponded to 100 *μ*M in Experiment 2 and was assumed to be 100-fold the clinically used dose [19]. Even following perfusion with 100-fold the clinically used dose of remimazolam or propofol, the intracellular Ca^2+^ concentration was not enhanced in response to the stimulant. Furthermore, the result of Experiment 3 showed that the EC_50_ values of remimazolam were 1.00 and 0.92 mM for cells not transfected with *RYR1* and those transfected with wild-type *RYR1*, respectively. Furthermore, the EC_50_ values of propofol were 1.09 and 1.05 mM for cells not transfected with *RYR1* and those transfected with wild-type *RYR1*, respectively. These EC_50_ values were much higher than the previously mentioned clinically used doses, and therefore, we considered that neither drug would raise the intracellular Ca^2+^ concentration with clinical use.

MH occurs when an MH-susceptible patient is exposed to a volatile inhalation anesthetic, and the severity of clinical symptoms is known to vary depending on the type of anesthetic [20]. Among volatile inhalation anesthetics, halothane causes fulminant MH symptoms, whereas those caused by sevoflurane or desflurane are known to manifest slower than that of halothane [21, 22]. In contrast, there are drugs such as succinylcholine that do not clearly cause MH on their own but intensely promote the pathology of MH induced by volatile inhalation anesthetics [23]. As described above, many aspects of the pathogenesis of MH are still unclear, and in some cases, there is a difference in the development of MH depending on the drug. Alternatively, a drug that does not cause MH by itself might promote the development of MH.

Remimazolam is a short-acting benzodiazepine sedative and anesthetic that is clinically used as a general anesthetic in Japan [24]. Remimazolam, like midazolam, is a benzodiazepine anesthetic and has been speculated to be potentially safe for use in MH-sensitive patients. However, we considered that it would be necessary to confirm the MH inducibility and stimulatory effect before clinical use in susceptible patients. Remimazolam did not enhance the Ca^2+^ dynamics of HEK-293 cells expressing mutant RYR1 induced in response to the RYR1 agonist caffeine. This suggests that remimazolam may not promote the development of MH in susceptible patients exposed to triggering agents. Consequently, remimazolam may be used as an alternative anesthetic in patients experiencing the onset of MH without promoting MH. Furthermore, remimazolam increased intracellular Ca^2+^ levels in a concentration-dependent manner, but this reaction did not differ in the presence or absence of RYR1. These results indicate that the increase in intracellular Ca^2+^ concentration induced by remimazolam was not an RYR1-mediated response.

The *RYR1* gene mutations used in our study were inserted in three regions known as “hotspots” in the *RYR1* gene where MH causative mutations are concentrated: the N-terminal (exons 2-17, p.Met35-p.Arg614), central (exons 39-46, p.Arg2163-p.Arg2458), and C-terminal (exons 90-104, p.Arg4136-p.Pro4973) regions [25]. In our study, we chose p.Thr84Met, p.Ser2345Arg, and p.Ala4894Thr from the N-terminal, center, and C-terminal, respectively. These *RYR1* mutations were confirmed to be associated with the pathogenesis of MH in previous studies [14–17], which we also confirmed in this study using CADD, MutationTaster, and PolyPhen-2 function prediction tools. Although many RYR1 mutations have been reported to meet the diagnostic criteria of the European Group of Malignant Hyperthermia (EMHG), our study validated only some of these mutations [26]. Furthermore, MH may have causes other than RYR1 and there are other possible *RYR1* mutations that have not yet been reported. Consequently, although we showed that remimazolam and propofol did not enhance the response to all known mutated RYR1, we cannot conclude that these agents are safe for all MH-susceptible patients. However, in our study, we found that remimazolam and propofol did not increase the responsiveness of RYR1 to caffeine including that of the mutated forms, which were identified to be sufficiently pathogenic. We believe that this result indicates that many potential *RYR1* mutations that were not validated in this study are not likely to enhance responsiveness to MH-inducing drugs following exposure to remimazolam or propofol [27, 28].

Propofol has been empirically safely used in patients with MH, and there are no clinical reports that it causes MH [6, 29]. In a study using myotubes cultured from muscle cells of MH patients, Migita et al. [19, 30] showed that clinically used concentrations of propofol do not increase intracellular Ca^2+^ levels, but high concentrations of propofol increase intracellular Ca^2+^ levels. Similar results were obtained in our study using HEK-293 cells. Specifically, the increase in intracellular calcium concentration induced by propofol is not mediated by RYR1, and the clinically used dose does not increase intracellular calcium concentrations. These results suggest that propofol can be safely used in MH patients with RYR1 dysfunction. Similar to propofol [31], local anesthetics can also be safely used in MH patients at clinically used doses, but exposure to high doses increases intracellular Ca^2+^ concentration [32]. A study comparing increases in intracellular Ca^2+^ concentrations following exposure of myotube cells of patients with and without MH susceptibility to local anesthetics speculated that the underlying mechanisms are not mediated by RYR1 [33]. As shown in these reports [19, 30–33] and by our data, even drugs that can be safely used in patients with MH can increase intracellular Ca^2+^ levels at high concentrations in *in vitro* experiments. The detailed mechanism by which remimazolam and propofol elevate intracellular Ca^2+^ levels requires further elucidation in future studies.

A limitation for this study is that we calculated EC_50_ using remimazolam at the dissolution limit concentration, but the dose-response curve did not show a plateau (see Figure 5). Although the EC_50_ of remimazolam presented in our research results in Table 5 is calculated by statistical software, it has a possibility of being underestimated than the actual value.

## 5. Conclusions

Remimazolam and propofol did not promote the caffeine-induced increase in intracellular Ca^2+^ levels in HEK-293 cells with mutated *RYR1* even following exposure to approximately 100-fold the dose used clinically. Furthermore, the increase in intracellular Ca^2+^ concentration caused by remimazolam or propofol was not a RYR1-mediated reaction. We conclude that remimazolam and propofol can be used in MH-susceptible patients with *RYR1*-mutation without causing MH and may be safely used as an alternative anesthetic to those that trigger MH when RYR1-mediated MH occurs.

## Figures and Tables

**Table 1:** The Fura-2 ratio (340/380 nm) of HEK-293 cells expressing wild-type or mutation RYR1 at resting state and at response to 10 mM and 20 mM caffeine. There was no statistically significant difference in the Fura-2 ratio at resting, response to 10 mM caffeine, or response to 20 mM caffeine between wild-type RYR1-expressed HEK-293 cells and mutated RYR1-expressed HEK-293 cells. There is no statistical difference between the response to 10 mM caffeine and the response to 20 mM caffeine in the Fura-2 ratio for any RYR1 (*P* value response of 10 mM caffeine vs. 20 mM caffeine is wild-type RYR1: 0.711667, p.Thr84Met: 0.777066, p.Ser2345Arg: 0.924708, and p.Ala4894Thr: 0.834281, respectively).

		*n*	Fura-2 ratio (340/380 nm)	*P* value
Resting	Wild-type	30	1.14 ± 0.20	
	p.Thr84Met	16	1.10 ± 0.15	0.333902
	p.Ser2345Arg	22	1.21 ± 0.16	0.058606
	p.Ala4894Thr	28	1.15 ± 0.19	0.882282

Response to 10 mM caffeine	Wild-type	30	1.99 ± 0.71	
	p.Thr84Met	16	1.96 ± 0.42	0.804009
	p.Ser2345Arg	22	1.81 ± 0.42	0.158114
	p.Ala4894Thr	28	2.08 ± 0.60	0.501581

Response to 20 mM caffeine	Wild-type	30	2.04 ± 0.78	
	p.Thr84Met	16	1.99 ± 0.43	0.673687
	p.Ser2345Arg	22	1.82 ± 0.42	0.101493
	p.Ala4894Thr	28	2.05 ± 0.59	0.949877

**Table 2:** Deleteriousness of transfected mutant ryanodine receptor 1 (RYR1). All three RYR1 mutations had high CADD scores (>20) and were predicted to be “disease causing” by MutationTaster and “possibly damaging” by PolyPhen-2.

Position	AA change	CADD	MT (probability score)	PP2 (probability score)
*RYR1* chr19: 38933074 C>T	p.Thr84Met	23.7	Disease causing (0.747)	Possibly damaging (1.000)
*RYR1* chr19: 38990282 C>A	p.Ser2345Arg	23.1	Disease causing (0.914)	Possibly damaging (0.954)
*RYR1* chr19: 39075616 G>A	p.Ala4894Thr	29.8	Disease causing (0.999)	Possibly damaging (0.999)

chr: chromosome; AA: amino acid; CADD: Combined Annotation-Dependent Depletion; MT: MutationTaster; PP2: PolyPhen-2.

**Table 3:** Median effective concentration (EC_50_) of caffeine in wild-type or mutant ryanodine receptor 1- (*RYR1-*) transfected cells. EC_50_ values were lower in mutant *RYR1*-transfected cells than in wild-type *RYR1*-transfected cells.

*RYR1*	*n*	Mean EC_50_ ± SD (mM)	95% CI (mM)	*P* value	Cohen's d
Wild-type	30	2.75 ± 0.81	2.46 to 3.10		
p.Thr84Met	16	1.71 ± 0.43	1.49 to 1.95	0.000238	1.60
p.Ser2345Arg	22	1.82 ± 0.56	1.58 to 2.08	0.000405	1.33
p.Ala4894Thr	28	1.39 ± 0.55	1.18 to 1.60	0.000031	1.97

SD: standard deviation; *n*: number of cells examined; 95% CI: 95% confidence interval; *P* values, vs. wild-type.

**Table 4:** Median effective concentration (EC_50_) values of caffeine in wild-type or mutant ryanodine receptor 1- (*RYR1-*) transfected cells perfused with remimazolam or propofol. EC_50_ values for caffeine in wild-type and all mutant *RYR1*-transfected cells were not significantly different with or without remimazolam (546 *μ*M) and propofol (100 *μ*M) perfusion.

*RYR1*	Perfusion solution	*n*	Mean EC_50_ ± SD (mM)	95% CI (mM)	*P* value
Wild-type	HBSS	30	2.75 ± 0.81	2.46 to 3.10	
	Remimazolam + HBSS	19	2.86 ± 1.19	2.31 to 3.51	0.764555
	Propofol + HBSS	25	2.76 ± 0.65	2.50 to 3.03	0.829001

p.Thr84Met	HBSS	16	1.71 ± 0.61	1.49 to 1.95	
	Remimazolam + HBSS	16	1.58 ± 0.63	1.26 to 1.91	0.623386
	Propofol + HBSS	16	1.65 ± 0.57	1.36 to 1.96	0.730406

p.Ser2345Arg	HBSS	22	1.82 ± 0.67	1.58 to 2.08	
	Remimazolam + HBSS	11	1.75 ± 0.77	1.27 to 2.21	0.472894
	Propofol + HBSS	13	1.48 ± 0.54	1.17 to 1.78	0.209642

p.Ala4894Thr	HBSS	28	1.39 ± 0.58	1.18 to 1.60	
	Remimazolam + HBSS	17	1.22 ± 0.61	0.92 to 1.48	0.594602
	Propofol + HBSS	17	1.13 ± 0.63	0.82 to 1.38	0.917061

SD: standard deviation; *n*: number of cells examined; 95% CI: 95% confidence interval; *P* values vs. Hank's balanced salt solution (HBSS) perfusion.

**Table 5:** Median effective concentration (EC_50_) of remimazolam or propofol in wild-type ryanodine receptor 1 (*RYR1*) or untransfected cells. EC_50_ values of remimazolam or propofol in wild-type *RYR1-*transfected and untransfected cells were not significantly different.

	*RYR1*	*n*	Mean EC_50_ ± SD (mM)	95% CI (mM)	*P* value
Remimazolam	Wild-type transfected cells	23	0.92 ± 0.34	0.82 to 1.10	0.914420
	Untransfected cells	17	1.00 ± 0.49	0.82 to 1.39	
Propofol	Wild-type transfected cells	49	1.05 ± 0.14	1.01 to 1.10	0.842608
	Untransfected cells	40	1.09 ± 0.07	1.06 to 1.11	

SD: standard deviation; *n*: number of cells examined; 95% CI: 95% confidence interval; *P* values, untransfected vs. wild-type *RYR1*-transfected cells.

## Data Availability

Requests for data will be considered by the corresponding author.
